# Molecular neurobiological clues to the pathogenesis of bipolar disorder

**DOI:** 10.1016/j.conb.2015.07.002

**Published:** 2016-02

**Authors:** Paul J Harrison

**Affiliations:** Department of Psychiatry, University of Oxford, Warneford Hospital, Oxford OX3 7JX, United Kingdom

## Abstract

Bipolar disorder is a serious psychiatric disorder, with a high heritability and unknown pathogenesis. Recent genome-wide association studies have identified the first loci, implicating genes such as *CACNA1C* and *ANK3.* The genes highlight several pathways, notably calcium signalling, as being of importance. Molecular studies suggest that the risk variants impact on gene regulation and expression. Preliminary studies using reprogrammed patient-derived cells report alterations in the transcriptome and in cellular adhesion and differentiation. Mouse models show that genes involved in circadian biology, acting via dopaminergic effects, reproduce aspects of the bipolar phenotype. These findings together represent significant advances in identification of the genetic and molecular basis of bipolar disorder, yet we are still far from an integrated, evidence-based understanding of its aetiopathogenesis.

## Highlights

• Bipolar disorder has a large genetic component, with many loci involved.
• Leading genes include *CACNA1C* and *ANK3*.
• There is genetic convergence on calcium signalling and other pathways.
• Genetic mouse models, and stem cells, are shedding light on pathogenesis.
• The advances have potential therapeutic benefits, but these remain distant.

**Current Opinion in Neurobiology** 2016, **36**:1–6

This review comes from a themed issue on **Neurobiology of disease**

Edited by **Dennis J Selkoe** and **Daniel R Weinberger**

For a complete overview see the Issue and the Editorial

Available online 25th July 2015

**http://dx.doi.org/10.1016/j.conb.2015.07.002**

0959-4388/© 2015 The Author. Published by Elsevier Ltd. This is an open access article under the CC BY license (http://creativecommons.org/licenses/by/4.0/).

## Introduction

Bipolar disorder (BD) is classically characterised by recurrent episodes of depression and elevated mood (mania), interspersed with periods of normal mood (euthymia) [1]. In reality, the clinical picture is more complex, with mixed mood states, residual cognitive dysfunction [2] and persistent mood instability during euthymia [3] often observed. During the mood swings, features of psychosis (delusions and hallucinations) may occur. The combination of mood and psychotic symptoms has contributed to uncertainty as to where BD sits within psychiatric classifications, and in particular its relationship to schizophrenia and to other mood disorders. BD affects 1–2% of the population, depending on the criteria used, and usually begins in adolescence or early adulthood. Morbidity is high, comorbidity with other psychiatric disorders common, and suicide occurs in at least 5%, contributing to a life expectancy that is reduced by over a decade. Lithium remains the gold standard for prophylaxis, with anticonvulsants, antipsychotics, and psychological treatments, also playing a key role in treatment [4].

BD is highly heritable, with estimates of over 80% from twin studies [5], yet understanding of its genetic basis, pathogenesis, and pathophysiology have remained frustratingly elusive, even by the standards of other psychiatric disorders. This partly reflects its inherent complexity and a relative dearth of research, but also difficulties in modelling the disorder in animals or cells. Fortunately, progress has recently been made in several domains. Those pertaining to the genetic and molecular aspects of BD are reviewed here. See [6] for a complementary review, focusing on the role of oxidative stress and cellular damage.

## Genomics of bipolar disorder

As with other psychiatric disorders, genome-wide association studies (GWAS) of single nucleotide polymorphisms (SNPs) indicate that the heritability of BD is attributable largely to multiple loci of small effect. Table 1 summarises the current genome-wide significant loci. See [5, 7] for recent reviews of BD genetics. Table 1 also notes the gene(s) implicated at each locus, but it is worth emphasising that the ‘causal’ gene, and true risk SNP or haplotype, at each locus remains unknown, hindering the interpretation of the GWAS signals, as is the case for all psychiatric disorders (see [8] for discussion; see below for discussion of some of the BD-implicated genes).

Other studies have begun to investigate genotype–phenotype relationships within BD, and between BD and other disorders. Within BD, genetic loci have been related to variables such as presence of psychotic symptoms, suicidality, and body mass index (e.g. [9]). Some of these analyses have revealed genome-wide associations, but the samples are inevitably smaller than for the overall BD-control comparisons; there is also the problem that detailed phenotyping is often not available. Reflecting the clinical and familial overlaps between BD and other psychiatric disorders, cross-disorder analyses have been carried out. These show substantial sharing of risk loci between BD and schizophrenia [10], and also significant commonalities with major depression, but little with autism or attention-deficit hyperactivity disorder [10, 11•]. Shedding new genetic light on an old debate, these data support the view that BD and schizophrenia are on a continuum, rather than being discrete disorders, as they have conventionally been classified [12].

Despite the progress, several major limitations and unknowns deserve mention, beyond the need to identify unambiguously the affected gene(s) at each locus. First, the causal genetic variant being tagged by the GWAS SNPs has not been identified. In the absence of known coding variants in linkage disequilibrium, the likelihood is that the risk SNPs alter gene regulation and expression [13]. There is preliminary evidence for this in terms of expression quantitative trait loci (eQTLs) [11•, 14•, 15] and for some of the individual genes, as discussed below. Second, the current BD GWAS signals explain only a fraction of the heritability. The source of the remainder is unknown, but likely includes many more independent loci, as well as gene–gene interactions (epistasis), gene–environment interactions, and a role for copy number variants (CNVs), although at present the data suggest that the latter are less important in BD than in schizophrenia or autism [12]. Other rare variants may also be involved, including those encoding calcium channel and GABA_A_ receptor subunits [16, 17•].

## Bipolar disorder genes and pathways

Two main approaches are being taken to understand the neurobiological consequences of the genes implicated to date in BD: bioinformatic identification of gene networks and pathways, and empirical studies of the expression and function of individual genes. The latter studies are also addressing the molecular basis of the genetic associations.

Pathway analyses using gene ontology and related methods are being carried out in various ways. A prominent category, for which there is convergent evidence, is calcium signalling, particularly voltage-gated calcium channels, identified in several datasets and using differing approaches. It is notable that calcium signalling was already hypothesised to be important in BD and its treatment [18, 19]; it was perhaps less expected that this enhancement of calcium channel genes applies similarly to schizophrenia, and also extends to other disorders [11^•^]. A range of other pathways are also highlighted in BD by studies which have combined GWAS data with gene expression data; these include hormonal regulation, second messenger systems and glutamatergic signalling [20^••^] as well as histone and immune pathways [21]. Each of these findings should be seen as provisional and in need of replication, not least given the inadequacies of gene ontology categories, and the incomplete information being fed into the analyses. Nevertheless, they do provide some of the first steps to a more meaningful integrative molecular classification and understanding of BD.

Complementing these approaches, the leading BD risk genes are being investigated individually, with the goal of understanding the neurobiology of the gene, the molecular basis of the disease association, and the functional impact of the putative risk variant. Three examples are given here.

*CACNA1C* encodes the L-type calcium channel Cav1.2 subunit and is arguably the best supported BD gene, especially if the various lines of prior evidence for altered calcium signalling in the disorder are taken into account [18, 19]. In addition to many studies reporting neuroimaging, cognitive and neurophysiological correlates of *CACNA1C* genotype, molecular studies have sought to show the proximal effect of the BD-associated SNPs. The risk haplotype, including the main SNP, rs1006737, resides within the large (300 kb) third intron, and is hence non-coding. Any functionality is likely to be via an effect on expression or splicing of *CACNA1C* – though, as with all such genetic associations, other explanations are possible (e.g. effects on antisense transcripts, non-coding RNAs, or distant genes [13]. The size and complexity of the gene (∼6.5 Mb, with at least 55 exons, and an unknown repertoire of transcript and protein isoforms) makes this a daunting task – especially since gene regulation is often different between tissues (as well as cell types and developmental stages), mandating the use of post mortem brain tissue as part of this approach [22]. In the first such study, in dorsolateral prefrontal cortex of over 250 individuals, Bigos *et al*. [23] showed that genotype affects *CACNA1C* mRNA abundance with risk homozygotes having highest expression. Results in induced neurons derived from fibroblasts show the same profile (see below). However, Gershon *et al*. [16] found the opposite result in cerebellum, and no genotype effect in parietal cortex, in separate brain series; these authors examined a larger range of *CACNA1C* SNPs, but had considerably smaller sample sizes, than [23]. Roussos *et al*. [15] also provide some evidence that the risk allele is associated with reduced *CACNA1C* expression in brain tissue, and in cell lines, and reveal a more complex relationship between *CACNA1C* gene structure and regulation beyond that conferred by rs1006737. Further studies are clearly required, taking into account the possibility of temporal and spatial specificity of effects, and genetic influences on splicing of the gene — to date only the overall *CACNA1C* transcript abundance has been assayed.

*ANK3* encodes ankyrin G, a scaffold protein involved in many cellular processes [24]. Like *CACNA1C*, *ANK3* is a large and complex gene (700 kb, with at least 44 exons and multiple protein isoforms). With regard to BD, perhaps the best known feature of *ANK3* is that it is located in the axon initial segment and at nodes of Ranvier, wherein it couples voltage-gated sodium channels to the cytoskeleton [25]. However, a recent study shows that *ANK3* is dispensable for this function [26], whilst Chang *et al*. [27] show that *ANK3* is expressed by oligodendrocytes, with anykyrin G abundant on the glial rather than axonal side of the nodes. These unexpected findings are complemented by reports showing that ankyrin G may have important additional localisations and functions. For example, Durak *et al*. [28] show that *ANK3* regulates the β-catenin/Wnt signalling pathway, already implicated in BD. Smith *et al*. [29^•^] identified a novel short *ANK3* isoform which is located in dendritic spines and regulates NMDA receptor-dependent plasticity. Interestingly, *ANK3* becomes enriched in this sub-synaptic fraction after chronic lithium treatment [30]. In BD, *ANK3* mRNA is increased in blood [31], but its expression has not been reported in brain in the disorder. The likelihood of isoform-selective functions of *ANK3* is complemented by preliminary evidence for differential expression of *ANK3* isoforms in human brain and their regulation by BD associated SNPs [32, 33]. The latter study [33] suggests that the risk allele at rs9804190 is associated with decreased *ANK3* mRNA, but other *ANK3* SNPs did not show significant effects on expression.

*ZNF04A* (zinc finger protein 804A) was originally identified as a schizophrenia risk gene but then found to be associated with a broader psychosis phenotype including BD. It is thought to encode a transcription factor, and regulates expression of various genes *in vitro* [34]. The best-established psychosis risk allele (rs1344706), located in the third intron, influences *ZNF804A* mRNA in foetal but not adult human brain [35], with the risk allele being associated with lower expression. Tao *et al*. [36^••^] replicated this observation in prefrontal cortex of a separate, large sample (*n* = 697) and showed that genotype impacts not on the full-length transcript, but on a newly identified, shorter *ZNF804A* mRNA isoform. The effect was again limited to foetal brain, although they also found a genotype-by-diagnosis interaction in their BD sample, with lower expression in patients homozygous for the risk allele. Complicating interpretation of the data, translation of the protein encoded by the short *ZNF804A* transcript has not been unambiguously demonstrated in human brain; moreover, its predicted sequence lacks the zinc finger domain of *ZNF804A*, and hence nothing is known about its potential function nor how it may be involved in BD pathogenesis.

In summary, these examples illustrate the depth and breadth of research which will be needed to move from statistical association of BD with a genomic locus to (a) identifying the affected gene(s), (b) clarifying its role in the pathogenesis of BD, and (c) determining the pathophysiological consequences of the risk SNP or haplotype.

## Animal models of bipolar disorder

All animal models of psychiatric disorders have inherent limitations, but BD has suffered particularly from the lack of a model which recapitulates its core feature, *viz*. spontaneous oscillations between manic-, euthymic-, and depressive-like phenotypes. However, there are several genetic mouse models which show manic-like behaviour and which have been used to explore its molecular and neural basis.

*SHANK3* point mutations and deletions are linked with autism, but not with BD. However, *Shank3*-overexpressing mice show manic-like behaviour, altered circadian rhythms, and a synaptic excitation/inhibition imbalance, via a pathway involving the Arp2/3 pathway and increases in F-actin [37]. Their behavioural phenotype could be normalised by one commonly used mood stabiliser, sodium valproate, but not by lithium.

The *Clock* Δ*19* mouse has a mutation in the *Clock* gene, a core regulator of the circadian system. The mouse was noted several years ago to have a diurnal manic-like behaviour, being hyperactive and with more reward-related and reduced depressive and anxiety behaviours during the light phase, but normal behaviour at night [38]. This manic-like daytime profile coincides with increased firing of dopamine neurons in the ventral tegmental area. Sidor *et al*. [39^•^], using a novel chronic optogenetic stimulation paradigm, provide convincing evidence that enhanced dopaminergic activity is causally related to the behaviour. They also show that the mechanism likely involves *Clock* acting as a transcriptional repressor of tyrosine hydroxylase, a gene involved in dopamine synthesis, suggesting that the *Clock* Δ*19* mutant may be less efficient in this respect. Interestingly, another mouse model has recently been reported which also involves a circadian gene (the nuclear receptor *REV-ERBα*) regulating dopamine regulation via tyrosine hydroxylase repression, and with similar mood-related behaviour [40^•^]. These two studies together argue for a renewed focus on circadian biology, and dopamine, in the core pathophysiology — and perhaps treatment — of BD and related psychiatric disorders.

The *Shank3* transgenic, *Clock* Δ*19* and *REV-ERBα* mice illustrate how genetic models can be used to identify synaptic, cellular and synaptic mechanisms underlying BD-relevant phenotypes. Animal models are also being used to investigate the function of BD-associated genes. For example, Leuiss *et al*. [41^••^] studied *ANK3* using heterozygous *Ank3*^*+/−*^ mice, and RNA interference to knock down *Ank3* in the dentate gyrus. Both manipulations led to decreased anxiety and increased motivation for reward, which were ameliorated by chronic lithium administration. Moreover the *Ank3*^*+/*−^ mice were more susceptible to depression-like behaviours after chronic stress, and had higher corticosterone levels, suggestive of enhanced stress reactivity.

## Modelling bipolar disorder in reprogrammed cells

Notwithstanding these developments, the lack of established animal models of bipolar disorder has encouraged the early adoption of induced pluripotent stem cell (iPSC) research into the field.

Chen *et al*. [42] generated iPSCs from fibroblasts of three BD patients and three controls, and differentiated them into cells with a forebrain neuronal phenotype. They reported alterations in the transcriptome of the BD-derived neurons (but not the iPSCs), including up-regulation of some receptor and ion channel transcripts and those specifying a ventral rather than dorsal telencephalic fate.

Wang *et al*. [43] took a different technical approach, directly trans-differentiating fibroblasts to neuron-like cells (induced neurons), from 12 BD patients (6 lithium responders, 6 non-responders) and 6 healthy controls. Using a label-free optical imaging method, they measured parameters related to cell growth, size, and adhesion. The main finding was that lithium responsive patients’ neurons showed greater adhesion compared to those of lithium non-responders. Cells from control subjects were intermediate between these two groups, and the BD group overall did not show differences from the controls. The study highlights that research using iPSCs, and derived neuronal-related cells may have value for understanding drug actions as well as disease pathophysiology.

Madison *et al*. [44] used a family-based paradigm, generating iPSC lines and then neural progenitor cells from two brothers with BD and their two unaffected parents. No differences were seen for the iPSCs between affected and unaffected members, but the neural progenitors showed several differences, including alterations in neurogenesis and in the expression of neuroplasticity-related genes including *Wnt* and ion channels.

Another experimental design is to compare cells derived from subjects on the basis of genotype, rather than by diagnosis, to examine genetic mechanisms. Yoshimizu *et al*. [45^••^] took fibroblasts to make induced neurons from 24 subjects genotyped for rs1006737, the risk-associated SNP in *CACNA1C*, and examined the expression and function of calcium channels. They found elevated *CACNA1C* mRNA, and greater calcium current density, in the neurons derived from risk homozygotes compared to heteroyzgotes and non-risk homozygotes. These data support the view that the risk variants of *CACNA1C* may involve a gain rather than a loss of function, and strengthen the rationale for L-type calcium channel antagonists as potential therapeutic agents in BD.

In summary, studies using reprogrammed cells are providing intriguing clues about the pathophysiology of neuronal-like cells derived from patients with BD, or related to its genetic risk. However, no clear pattern or convergence of findings has yet emerged, and much remains to be done in terms of detailed phenotyping, and particularly in the scaling up of experiments to provide robust and generalisable results.

## Conclusions

Recent findings have made inroads into revealing the genetic and molecular basis of BD, and have provided support for the long-suspected (but still poorly characterised) role of calcium signalling in the disorder, as well as implicating pathways and processes which were less anticipated. There is a long road ahead in terms of identifying the pathophysiological implications of the risk genes, and understanding how their functioning is impacted by the risk variants. At present, the effects appear to be mediated through altered gene expression and thence function, with some evidence that specific isoforms in each gene may be important. The challenges and opportunities ahead are both empirical and bioinformatic, and both will expand substantially with the forthcoming GWAS from the Psychiatric Genomics Consortium involving 20,000 BD cases and 28,000 controls [7] which will no doubt reveal many more loci. In tandem with the ongoing efforts to understand the molecular mechanism of action of lithium and other mood stabilisers [46, 47, 48], there are grounds for cautious optimism that transformative advances in therapies for BD will soon be possible. These are long overdue.

## Conflicts of interest

No relevant conflicts of interest.

## References and recommended reading

Papers of particular interest, published within the period of review, have been highlighted as:

• of special interest
•• of outstanding interest

## Figures and Tables

**Table 1:** Genome-wide significant loci for bipolar disorder.

Locus	Implicated gene(s) and symbol(s)
*Genome-wide significant in BD*	
10q21.2	Ankyrin 3 (*ANK3*)
12p13.3	Calcium channel, voltage-dependent, L-type, alpha 1C subunit (*CACNA1C*)
11q14.1	Teneurin transmembrane protein 4 (*TENM4*, formerly known as *ODZ4*)
19p12	Neurocan (*NCAN*)
6q25.2	Spectrin repeat containing, nuclear envelope 1 (*SYNE1*)
3p22.2	Tetratricopeptide repeat and anykrin repeat containing 1 (*TRANK1*)
5p15.31	Adenylate cyclase 2 (*ADCY2*)
6q16.1	MicroRNA 2113 (*MIR2113*); POU class 3 homeobox 2 (*POU3F2*; formerly known as *OTF7*)
10q24.33	Arsenite methyltransferase (*AS3MT*)

*Genome-wide significant in BD* + *schizophrenia (combined)*	
2q32.1	Zinc finger protein 804A (*ZNF804A*)
3p21.1	Inter-alpha-trypsin inhibitor heavy chain 3 (*ITIH3*); inter-alpha-trypsin inhibitor heavy chain 4 (*ITIH4*)
16p11.2	Mitogen-activated protein kinase 3 (*MAPK3*)

*Genome-wide significant in BD* + *unipolar depression (combined)*	
3p21	Polybromo 1 (*PBRM1*)

Adapted from [5, 7, 49•].
